# Primary Care Pre-Visit Electronic Patient Questionnaire for Asthma: Uptake Analysis and Predictor Modeling

**DOI:** 10.2196/19358

**Published:** 2020-09-18

**Authors:** Andrew Kouri, Janet Yamada, Joanna E M Sale, Sharon E Straus, Samir Gupta

**Affiliations:** 1 Division of Respirology, Department of Medicine St. Michael’s Hospital, Unity Health Toronto Toronto, ON Canada; 2 Daphne Cockwell School of Nursing, Ryerson University Toronto, ON Canada; 3 Li Ka Sing Knowledge Institute St. Michael's Hospital, Unity Health Toronto Toronto, ON Canada; 4 Department of Medicine, Division of Geriatrics St. Michael's Hospital, Unity Health Toronto Toronto, ON Canada

**Keywords:** electronic questionnaire, tablet, mHealth uptake, asthma, modeling

## Abstract

**Background:**

mHealth tablet-based interventions are increasingly being studied and deployed in various health care settings, yet little knowledge exists regarding patient uptake and acceptance or how patient demographics influence these important implementation metrics.

**Objective:**

To determine which factors influence the uptake and successful completion of an mHealth tablet questionnaire by analyzing its implementation in a primary care setting.

**Methods:**

We prospectively studied a patient-facing electronic touch-tablet asthma questionnaire deployed as part of the Electronic Asthma Management System. We describe tablet uptake and completion rates and corresponding predictor models for these behaviors.

**Results:**

The tablet was offered to and accepted by patients in 891/1715 (52.0%) visits. Patients refused the tablet in 33.0% (439/1330) visits in which it was successfully offered. Patients aged older than 65 years of age (odds ratio [OR] 2.30, 95% CI 1.33-3.95) and with concurrent chronic obstructive pulmonary disease (OR 2.22, 95% CI 1.05-4.67) were more likely to refuse the tablet, and those on an asthma medication (OR 0.55, 95% CI 0.30-0.99) were less likely to refuse it. Once accepted, the questionnaire was completed in 784/891 (88.0%) instances, with those on an asthma medication (OR 0.53, 95% CI 0.32-0.88) being less likely to leave it incomplete.

**Conclusions:**

Older age predicted initial tablet refusal but not tablet questionnaire completion, suggesting that perceptions of mHealth among older adults may negatively impact uptake, independent of usability. The influence of being on an asthma medication suggests that disease severity may also mediate mHealth acceptance. Although use of mHealth questionnaires is growing rapidly across health care settings and diseases, few studies describe their real-world acceptance and its predictors. Our results should be complemented by qualitative methods to identify barriers and enablers to uptake and may inform technological and implementation strategies to drive successful usage.

## Introduction

The use of information technology in health care has proliferated rapidly over the past 20 years. For example, patient-facing handheld touchscreen computers (tablets)—a form of mobile health (mHealth)—are increasingly being used and studied across a range of clinical settings [1-6]. Tablets are particularly well suited for collecting information directly from patients and have several potential advantages over traditional paper-based methods, including increased efficiency, facilitated data entry, greater data security, and ease of data transport [7,8]. Tablet-based patient questionnaires are also perceived favorably by patients, can promote greater patient-centered care, and are cost efficient [7,9]. Given these advantages, many clinicians and health care organizations are now integrating tablets into care. In Canada, 3500 physicians are already using electronic medical record–integrated tablet-based mHealth questionnaires, and the Canadian Medical Association has partnered with technology providers to further promote the use of tablets [10].

Patient uptake is critical to realizing the health system benefits of tablet-based questionnaires. However, despite broad interest and increasing use of tablets, there is limited research evaluating real-world mHealth tablet uptake and acceptability. Certain groups, such as older patients and those with lower educational attainment, may have disproportional barriers to accepting and using tablets [6,11] and a higher risk of experiencing technology-induced medical errors [12]. Accordingly, further research is required to understand the differences in uptake across demographics and the reasons for these differences, with a view toward improving implementation processes and technologies to enable broad acceptance.

We recently completed a prospective study of a chronic disease management tool called the Electronic Asthma Management System (eAMS) [13], which consisted of a patient-facing electronic touch-tablet asthma questionnaire for use in waiting rooms, that directly informed a provider-facing computerized clinical decision support system. Herein, we report on the implementation of this tablet-based questionnaire, including patient uptake, completion rates, and predictors of these metrics, in a real-world primary care setting.

## Methods

### Questionnaire Development

The eAMS questionnaire was created as a web-based app for Apple iPad and requires approximately 10 minutes to complete [14]. Questions were designed to (1) document current asthma control criteria, (2) identify current asthma medications used and adherence to these medications, and (3) collect information required to create a personalized self-management asthma action plan. Questionnaire data were transferred to a computerized clinical decision support system, which provided primary care clinicians with patients’ asthma control status, evidence-based recommendations for medication adjustments, and a prepopulated asthma action plan, integrated into the electronic medical record in real time [15]. A questionnaire prototype was developed by asthma and knowledge translation experts in our research group by applying evidence-based electronic questionnaire design principles [16]. After pilot testing in a convenience sample of clinicians and researchers, the prototype’s content and usability were iteratively refined using a rapid-cycle design process with serial focus groups (in total, 20 patients with asthma), followed by summative qualitative and quantitative analyses [14,15]. The questionnaire was designed and tested for use across a range of ages (with 30% of included participants aged older than 60 years of age), disease severities, and technological experience [14,15]. The system had a final System Usability Scale score of 84.2. The reported mean System Usability Scale score for web-based systems is 68, with a score of 84.2 corresponding to a subjective usability rating of excellent and a 95th percentile rank. The high System Usability Scale score was supported by favorable Likert-scale question responses and qualitative findings [14].

### Study Design and Population

The eAMS patient questionnaire and clinical decision support system were tested in a 1-year prospective interrupted time-series design study across 3 primary care sites in Ontario, Canada [13]. Detailed questionnaire uptake and completion data were captured at 2 of these clinics. We included patients aged older than 16 years, with a physician diagnosis of asthma (these patients were identified according to an electronic medical record search algorithm validated in this population and with the same electronic medical records used in study sites [17]), and with an asthma medication prescription in the prior year (but not necessarily using asthma medication at the time of the study). We excluded patients who were pregnant (as the questionnaire was designed in part to inform creation of a physician-delivered asthma action plan, and there is limited evidence for asthma action plan use during pregnancy [13]), had cognitive or language difficulties, had a life expectancy of <1 year, or had been on a medication for chronic obstructive pulmonary disease in the prior year [17]. The study was approved by St. Michael’s Hospital and Hamilton Integrated research ethics boards.

### Questionnaire Implementation

Each day, an on-site research assistant at each site used a database query to identify all eligible patients who were booked to see a physician or nurse practitioner (excluding visits exclusively for administration of injections such as the flu vaccine). The research assistant was tasked with identifying patients for questionnaire completion in the clinic waiting room and inviting them to complete the questionnaire on 1 of 2 available tablet devices, prior to their appointment. Patients who had completed the questionnaire within the prior 28 days were not asked to repeat it.

### Data Collection

Research assistants recorded tablet uptake and completion information for each eligible patient in a standardized Excel (Microsoft Inc) spreadsheet. This information included whether or not the tablet was given to the patient and the reason it was not given (if applicable) and whether the questionnaire was fully completed and reasons for noncompletion (if applicable, though patients were not required to provide a specific reason if they refused to accept the tablet or complete the questionnaire). We also collected demographic and clinical data including age, sex, smoking status, asthma medication prescriptions, history of emergency room visits or hospitalizations for asthma (since 2003), the presence or absence of prior physician documentation of a diagnosis of asthma or chronic obstructive pulmonary disease, and whether the patient’s asthma was currently under control through an electronic chart audit.

### Outcomes

The primary outcomes were tablet questionnaire uptake—the proportion of visits in which the tablet questionnaire was offered to and accepted by patients—and tablet questionnaire completion—the proportion of visits in which the questionnaire was completed by patients when uptake was successful. Secondary outcomes were the reasons for failed uptake and the reasons for failed completion. Among cases where the tablet questionnaire was offered to patients, we also sought to determine if tablet refusal and questionnaire noncompletion were influenced by the following patient-level variables (established a priori, based on clinical relevance): age, sex, smoking status, asthma medication prescription, physician-documented diagnosis of asthma or chronic obstructive pulmonary disease, history of emergency room visit or hospitalization related to asthma, and current asthma control status. Finally, we evaluated the proportion of patients who accepted the tablet questionnaire at least once who were willing to accept it on a subsequent occasion, as well as predictors of accepting it on a subsequent occasion. We compared key outcomes between clinical sites.

### Data Analysis

We present summary statistics for tablet uptake and completion rates (by patient visit), and reasons for uptake and completion failures. We used chi-square and Fisher exact tests to compare results between sites. We then constructed a logistic regression model to evaluate the influence of patient characteristics on the odds of tablet refusal and questionnaire noncompletion. In order to account for repeated measures at the patient level and clustering at the clinic level, generalized estimating equations were used [18]. Though the patient visits were not predetermined by the study investigators in this real-world study and follow-up was irregular, generalized estimating equations remain appropriate for analysis as the outcomes of interest were not clinical in nature, thus outcome-dependent follow-up was unlikely [19,20]. In order to ensure that this assumption was valid, another logistic regression model was constructed as a sensitivity analysis, modeling tablet refusal on only first patient visits, removing any potential residual confounding effects of repeated visits. Model performance was assessed by examining the concordance (C) statistic. At the patient level, we present summary statistics for the number of times each patient accepted the tablet questionnaire. We constructed a logistic regression model identifying predictors of refusing the tablet on the second occasion after acceptance on the first occasion. Additional available predictors for this patient-level analysis were time between completion of the first questionnaire and next time being offered it, and whether or not the questionnaire was fully completed on the first occasion it was accepted. All statistical analyses were performed using SAS (university edition; SAS Institute). A *P* value<.05 was statistically significant.

## Results

### Primary Outcome: Tablet Questionnaire Uptake

There were 612 eligible patients who made 1715 eligible visits during the study period (visits per patient: median 2, IQR 1-4) (Table 1). The tablet questionnaire was successfully offered and accepted in 891/1715 visits, resulting in an overall uptake of 52.0%. Failed uptake was due to the tablet not being offered in 385/1715 (22.5%) visits and not being accepted in 439/1330 (33.0%) visits in which it was offered. The tablet questionnaire was completed in 784/891 (88.0%) visits in which uptake (tablet offered and accepted) was successful (Table 1).

**Table 1:** Tablet uptake and completion.

			All, n (%)		Clinic 1, n (%)		Clinic 2, n (%)
Patients			612		437		175
Eligible visits			1715		1348		367
**Tablet offered to patient**			1715 (100)		1348 (100)		367 (100)
	Yes	1330 (77.6)		1084 (80.4)		246 (67.0)	
	No	385 (22.5)		264 (19.6)		121 (33.0)	
**Tablet accepted by patient after being offered**			1330 (100)		1084 (100)		246 (100)
	Yes	891 (67.0)		699 (64.5)		192 (78.0)	
	No	439 (33.0)		385 (35.5)		54 (22.0)	
**Questionnaire completed after tablet offered and accepted by patient**			891 (100)		699 (100)		192 (100)
	Yes	784 (88.0)		618 (88.4)		166 (86.5)	
	No	107 (12.0)		81 (11.6)		26 (13.5)	

### Secondary Outcomes: Reasons for Tablet Uptake and Completion Failures

Both technology-related and logistical issues prevented the tablet from being offered to patients. Most patients who refused the tablet did not provide a reason. Once accepted, patients failed to complete the questionnaire for a variety of reasons, including aborting the questionnaire due to difficulties in completing it, technology-related failures, or clinicians calling patients in from the waiting room (Table 2).

**Table 2:** Reasons for failed tablet uptake and completion.

Reasons		All visits, n (%)	Clinic 1 visits, n (%)	Clinic 2 visits, n (%)	*P* value
**Reasons tablet not offered to patients**		385 (100)	264 (100)	121 (100)	<.001^a^
	Patient missed by research assistant	307 (79.7)	215 (81.4)	92 (76.0)	
	Technology-related issue^b^	48 (12.5)	38 (14.4)	10 (8.3)	
	Both tablets already in use	12 (3.1)	7 (2.7)	5 (4.1)	
	Not documented	18 (4.7)	4 (1.5)	14 (11.6)	
**Reasons tablet not accepted by patients when offered**		439 (100)	385 (100)	54 (100)	.70^c^
	Patient refusal (denies asthma diagnosis)	54 (12.3)	46 (12.0)	8 (14.8)	
	Patient refusal (indicates too difficult to use)	24 (5.5)	22 (5.7)	2 (3.7)	
	Patient refusal (no reason provided)	351 (80.0)	307 (79.7)	44 (81.5)	
	Caregiver refusal	10 (2.3)	10 (2.6)	0 (0.0)	
**Reasons questionnaire not completed when tablet accepted**		107 (100)	81 (100)	26 (100)	<.001^c^
	Patient aborted (no reason provided)	46 (43.0)	45 (55.5)	1 (3.9)	
	Patient aborted (indicates too difficult to complete)	17 (15.9)	8 (9.9)	9 (34.6)	
	Patient aborted (started, then denied asthma diagnosis)	11 (10.3)	6 (7.4)	5 (19.2)	
	Technology-related issue^b^	5 (4.7)	2 (2.5)	3 (11.5)	
	Clinician aborted (called patient in prior to finishing)	28 (26.1)	20 (24.7)	8 (30.8)	

^a^Chi-square test was used.

^b^Technology-related issues included poor Wi-Fi or network connectivity and tablet software or hardware malfunctions.

^c^Fisher exact test was used.

### Predictors of Tablet Questionnaire Refusal

Among the 1330 visits in which the tablet was successfully offered, we were able to match 1026 visits (77.1%) to patient characteristics for modeling. Among these 1026 visits, the tablet was accepted on 679 (66.2%) occasions and refused on 347 (33.8%) occasions. In the multivariable model, being 65 years and older was associated with the greatest odds of tablet questionnaire refusal (odds ratio [OR] 2.30, 95% CI 1.34-3.95). Having a previously documented diagnosis of chronic obstructive pulmonary disease was also associated with increased odds of refusal (OR 2.22, 95% CI 1.05-4.67). Conversely, a current prescription for an asthma medication was associated with lower odds of refusal (OR 0.55, 95% CI 0.30-0.99). The model demonstrated good predictive ability, with a C statistic of 0.67. All predictors are shown in Table 3. Patient asthma control level was missing in 40% of visits (a known care gap [21]) and was therefore excluded from the model.

In the multivariable model constructed with only the data from 451 first patient visits, the tablet was accepted on 407 (90.2%) occasions and refused on 44 (9.8%) occasions. In order to limit the potential for overspecification, only the covariates meeting or approaching statistical significance in the original model or those that were thought to be clinically important were included in the model, which included age ≥65 years, sex, current asthma medication prescription, physician-documented diagnosis of chronic obstructive pulmonary disease, and clinic site. In this sensitivity analysis, being 65 years of age or older was again associated with the greatest odds of tablet questionnaire refusal (OR 2.79, 95% CI 1.39-5.59), controlling for other confounders. Prescription of an asthma medication and physician diagnosis of chronic obstructive pulmonary disease also remained statistically significant predictors. Full results from the sensitivity analysis can be found in Multimedia Appendix 1.

**Table 3:** Predictors of tablet refusal.

Patient-level descriptors		Visits (n=1026), n (%)	Tablet refused (proportion of visits), n (%)	Odds ratio (95% CI)
**Age**				
	<65 years old	750 (73.1)	208 (27.7)	reference
	≥65 years old	276 (26.9)	139 (50.4)	2.30 (1.33-3.95)
**Sex**				
	Male	263 (25.6)	81 (30.8)	reference
	Female	763 (74.4)	266 (34.9)	1.18 (0.68-2.06)
**Current asthma medication prescription**				
	No asthma medications	218 (21.3)	90 (41.3)	reference
	Any asthma medication	808 (78.7)	257 (31.8)	0.55 (0.30-0.99)
**Smoking status**				
	Lifelong nonsmoker	489 (47.7)	161 (32.9)	reference
	Current or former smoker	432 (42.1)	136 (31.5)	0.84 (0.48-1.46)
	Smoking history not documented	105 (10.2)	50 (47.6)	1.74 (0.79-3.83)
**Physician-documented diagnosis of COPD^a^**				
	Absent	887 (86.5)	274 (30.9)	reference
	Present	139 (13.5)	73 (52.5)	2.22 (1.05-4.67)
**Physician-documented diagnosis of asthma**				
	Absent	310 (30.2)	106 (34.2)	reference
	Present	716 (69.8)	241 (33.7)	1.13 (0.68-1.89)
**Prior ER^b^ visit or hospitalization for asthma**				
	No	951 (92.7)	330 (34.7)	reference
	Yes	75 (7.3)	17 (22.7)	0.59 (0.15-2.32)
**Clinic site**				
	Site 2	169 (16.5)	35 (20.7)	reference
	Site 1	857 (83.5)	312 (36.4)	1.63 (0.79-3.35)

^a^COPD: chronic obstructive pulmonary disease.

^b^ER: emergency room.

### Predictors of Failure to Complete Questionnaire After Accepting Tablet

Among the 891 visits in which patients accepted the tablet, we were able to match 706 visits (79.2%) to patient characteristics for modeling. After removing 27 visits where the questionnaire was not completed for reasons outside of the patient’s control (such as the patient being called in prior to finishing or technology-related issues), 679 visits remained, among which the questionnaire was completed on 605 (89.1%) occasions and not completed on 74 (10.9%) occasions. In the multivariable model considering these visits, use of asthma medications was the only statistically significant predictor and was associated with lower odds of failing to complete the questionnaire (OR 0.53, 95% CI 0.32-0.88). The logistic model demonstrated adequate predictive ability with a C statistic of 0.64. All predictors are shown in Table 4. Asthma control level was missing in 40% of visits and was therefore excluded from the model.

**Table 4:** Predictors of failure to complete the questionnaire after tablet acceptance.

Patient-level descriptors		Visits (n=1026), n (%)	Tablet refused (proportion of visits), n (%)	Odds ratio (95% CI)
**Age**				
	<65 years old	542 (79.8)	53 (9.8)	reference
	≥65 years old	137 (20.2)	21 (15.3)	1.48 (0.88-2.49)
**Sex**				
	Male	182 (26.8)	24 (13.2)	reference
	Female	497 (73.2)	50 (10.1)	0.77 (0.45-1.31)
**Current asthma medication prescription**				
	No asthma medications	128 (18.8)	22 (17.2)	reference
	Any asthma medication	551 (81.2)	52 (9.4)	0.53 (0.32-0.88)
**Smoking status**				
	Lifelong nonsmoker	328 (48.3)	35 (10.7)	reference
	Current or former smoker	296 (43.6)	36 (12.2)	1.00 (0.60-1.67)
	Smoking history not documented	55 (8.1)	2 (3.6)	0.48 (0.15-1.62)
**Physician-documented diagnosis of COPD^a^**				
	Absent	613 (90.3)	62 (10.1)	reference
	Present	66 (9.7)	12 (18.2)	1.90 (0.96-3.75)
**Physician-documented diagnosis of asthma**				
	Absent	204 (30.0)	30 (14.7)	reference
	Present	475 (70.0)	44 (9.3)	0.79 (0.47-1.34)
**Prior ER^b^ visit or hospitalization for asthma**				
	No	621 (91.5)	70 (11.3)	reference
	Yes	58 (8.5)	4 (6.9)	0.66 (0.24-1.81)
**Clinic site**				
	Site 2	134 (19.7)	15 (11.2)	reference
	Site 1	545 (80.3)	59 (10.8)	0.88 (0.47-1.67)

^a^COPD: chronic obstructive pulmonary disease.

^b^ER: emergency room.

### Willingness to Accept the Tablet on More Than One Visit

Of the 612 eligible patients in the study, 507 (82.8%) accepted the tablet at least once, 208 (34.0%) accepted the tablet at least twice, 84 (13.7%) accepted the tablet at least three times, and 45 (7.4%) accepted it four or more times.

Among the 507 patients who accepted the tablet at least once, 248 (48.9%) were offered the tablet a second time, among whom 208 (83.9%) accepted it.

In a multivariable model including age older than 65 years, sex, study site, time between accepting the first questionnaire and the next time it was offered, and whether or not the patient completed the questionnaire on the occasion in which it was first accepted, the only statistically significant predictor of refusing to accept the questionnaire a second time after accepting it the first time was noncompletion during the first attempt. Not having completed the questionnaire on the first instance had an odds ratio of 5.73 (95% CI 2.31-14.21) for refusing the questionnaire on the second eligible opportunity (for full details of model, see Multimedia Appendix 1).

## Discussion

### Principal Results

Our analysis revealed that a simple tablet-based questionnaire for use in waiting rooms was successfully offered and accepted by patients in only 52.0% (891/1715) and completed in only 45.7% (784/1715) of eligible visits in primary care over a period of 1 year. Although use of tablet-based questionnaires is growing rapidly in health care, few studies have explored their use in primary care settings, and knowledge about predictors of their real-world uptake is still limited. Although other investigators have reported on patient surveys of tablet acceptability [6,11,22-29], to our knowledge, ours is the first study to examine tablet questionnaire uptake and completion rates in a primary care setting *and* to quantitatively identify predictors of uptake and successful completion.

The first barrier encountered to tablet questionnaire uptake was failure of the study site research assistants to offer the tablet to eligible patients. This accounted for 17.9% of instances of failed uptake (307/1715). The main reason for this was research assistants missing an eligible patient in the busy clinic environments. The number of missed patients differed between the 2 clinic sites, suggesting that clinic-specific factors such as patient flow, clinic staff engagement, and physical layout may impact successful tablet implementation. This finding aligns with the results of previous systematic reviews [30] of eHealth implementation in general, which have highlighted the importance of good fit between eHealth systems and clinic workflows. One strategy to mitigate this workflow barrier could be to tie tablet distribution to the patient registration process upon arrival, such that the same personnel member who registers the patient (eg, a clinic receptionist) distributes the tablet. However, identifying appropriate patients, distributing, and managing tablets, including collecting and disinfecting between patients [14], may not be feasible for a clinic receptionist. Overall, our findings support the notion that strategies to successfully deliver tablets need tailoring to each clinic environment and that prelaunch workflow analysis [31] and pilot testing may be beneficial.

Of note, technology issues such as Wi-Fi or network failures and hardware or software issues prevented tablet uptake in only 48/1715 (2.8%) cases and completion in only 5/891 (1.0%) cases (Table 2). Though technology failures are recognized barriers to eHealth implementation success [30], they were only minor contributors to failed uptake in our study. This trend is likely to continue as smartphone use becomes more ubiquitous [32], and electronic questionnaires can instead be offered on patients’ personal devices.

The second major barrier to uptake was patient refusal to accept the tablet questionnaire, which occurred in 33.0% (439/1330) of visits (Table 1). Refusal levels did not differ between clinics, suggesting that this barrier is mediated mostly by patient-level factors (Table 2). In our multivariable model, patient age older than 65 years of age was the strongest predictor of tablet refusal (Table 3). This finding could be related to known decreased acceptance and comfort-levels with mobile technology among older adults [33,34] and may also reflect a decreased willingness among older adults to adopt newer paradigms of care in which patients are expected to play a more active role [35-37]. Previous researchers have shown somewhat conflicting results with regard to the influence of older age on tablet usage. Some authors have shown that increasing age is associated with decreased preference for tablet questionnaires over paper version [27], increased time needed to complete tablet questionnaires [38], and increased difficulty using tablets [6,11,22,28,29]. However, others have reported no significant influence of increasing age on tablet questionnaire usability, feasibility, and preference over paper versions [4,5,25,39]. Interestingly, being older than 65 years of age was not associated with failure to complete the tablet questionnaire in patients who accepted it. This finding may reflect the fact that our questionnaire development process included older patients (30% were 60 years of age or older) [14], and thus if the initial acceptance barrier was overcome, the electronic questionnaire had good usability across age ranges. These findings underscore the importance of including older individuals in the development process of mHealth tools but also that optimal tool usability across age ranges alone is not sufficient to drive uptake, and other implementation strategies to engage older individuals need to be considered as well.

Patients with a physician diagnosis of chronic obstructive pulmonary disease were also more likely to refuse the tablet questionnaire (Table 3). There is often a degree of overlap between asthma and chronic obstructive pulmonary disease in patients with airways disease, and some patients who experience this overlap may identify more as having chronic obstructive pulmonary disease than asthma [40,41]. It isn’t clear currently if and how patients with asthma–chronic obstructive pulmonary disease overlap engage differently with mHealth interventions targeted at either condition, as this group is poorly studied, but our findings suggest that there may be barriers to mHealth acceptance targeted specifically at one of their codiagnoses.

The only statistically significant predictor in both models was having a prescription for an asthma medication, which was associated with lower odds of both tablet questionnaire refusal and noncompletion (refusal: OR 0.55, 95% CI 0.30-0.99; noncompletion: OR 0.53, 95% CI 0.32-0.88). The fact that patients using asthma medications were less likely to refuse and more likely to complete the tablet questionnaire may reflect a greater interest in and willingness to invest in disease management among patients with more active disease (ie therapy-meriting disease). It is also of note that a fair proportion of both tablet refusals (54/439, 12.3%) and failed questionnaire completions (11/107, 10.3%) were due to patients denying that they had asthma (Table 2). Although use of a probabilistic electronic medical record search algorithm may have incorrectly identified some patients as having asthma [17], it is also likely that some patients with asthma who are not actively taking asthma medications do not self-identify as currently having asthma. Although health status–based differences in mHealth uptake have not previously been reported, research into how an individual’s health status influences desire for autonomy and preference for actively participating in care provides conflicting evidence across different patient populations and care settings [37,42,43]. In asthma in particular, there is recent evidence to suggest that there is an overall high level of desire among patients for autonomy and collaborative decision making regardless of disease severity [44,45]. However, how this relates to mHealth and tablet use is not clear and warrants further study.

The barriers and predictors discussed above illuminate important practical factors requiring consideration when implementing a tablet-based questionnaire. Many are likely relevant to any mHealth questionnaire, including smartphone questionnaires. Ideally, strategies to optimize usage of such tools will involve formal measurement of barriers and enablers and a theory-based approach to overcoming and leveraging these, respectively. To this end, we used complementary quantitative and qualitative research methods to specifically identify barriers and enablers to mHealth questionnaire uptake, through a Theoretical Domains Framework analysis [46]. We will use these findings to match specific behavior-change strategies to the identified barriers and enablers, in order to inform system changes and implementation strategies that will drive usage.

Encouragingly, we found that when the tablet was successfully offered to and accepted by patients, overall, questionnaire completion occurred in 88% (784/891) of cases. This finding corresponds with our previous study’s findings [14,15] of high questionnaire usability and further validates the importance of patient engagement and iterative questionnaire design with a focus on both content and usability. The unexpected finding that older patient age did not negatively influence tablet questionnaire completion underscores this importance further and supports our decision to deliberately include patients from a wide age range in our tablet questionnaire development process. This finding provides an important addition to the literature, as it implies that provided initial acceptance barriers can be overcome, optimizing mobile interface usability across patient demographics can lead to successful engagement in patient populations that are traditionally thought to be more difficult to reach (for example, older adults). This finding has implications for the design of future implementation strategies for mobile technology in older adults.

Finally, we assessed patients’ willingness to complete tablet questionnaires on more than one occasion. The vast majority (208/248, 83.9%) of patients who accepted the tablet the first time accepted it again on the second opportunity. This suggests that once initial buy-in is achieved with patients, the majority see sufficient value in the process to continue to participate. The only significant predictor of refusing the tablet on the second occasion after having accepted it on the first was not having completed the tablet fully on the first attempt (OR 5.73, 95% CI 2.31-14.21). Programs considering routine use of tablet questionnaires should ensure that patients have adequate time and support to complete the questionnaire on the first attempt, as our findings suggest that a failed first attempt significantly deters future participation.

### Limitations

Our study has some limitations. Although asthma patients were identified by a validated electronic medical record–based search algorithm [17] and were further required to have been prescribed an asthma medication in the prior year, some patients without asthma were likely erroneously identified due to imperfect algorithm specificity. Patients denied an asthma diagnosis in 65 visits, but this represented only 3.8% of the total 1715 visits and is unlikely to have influenced our main conclusions. We were unable to include about 20% of visits in our predictive models due to an inability to match those visits to patient characteristics. This resulted from the fact that patient characteristics were collected independently, and at a separate point in the study, whereby certain patients who were offered the questionnaire did not have their clinical characteristics recorded. This process can be assumed to have occurred at random; there was no difference in the tablet questionnaire acceptance level between patients who had full data and the whole cohort. We were also unable to obtain real-time qualitative patient feedback on tablet use during the study, as we designed our study to not interfere with clinic workflow as much as possible. However, we have completed an accompanying qualitative analysis of barriers and enablers to mobile health questionnaire acceptance and uptake in patients with asthma using this tablet questionnaire. It should also be noted that tablet questionnaires can be delivered via various operating systems and tablet sizes. Although we would not expect metrics related to offering the patients the tablet, or willingness of patients to accept the tablet to be affected by such differences, our findings regarding tablet completion rates may not be generalizable to other hardware and operating system types. Finally, as the denominator for our primary analysis was patient visits, there is the possibility that repeated measures from patients being offered the tablet on multiple occasions might have biased our analysis. To account for this, we used generalized estimating equations, which enable a population average model that adjusts for the correlated nature of repeated measurements. We also replicated our main results in a sensitivity analysis which included only a single visit for each patient.

### Conclusion

In summary, we found that an electronic tablet-based asthma questionnaire implemented in a primary care waiting room setting had an overall uptake of only 52.0% (891/1715), even with involvement of dedicated research personnel. Patients refused to accept or to complete the tablet for various reasons. In particular, older age was an important predictor of tablet refusal, despite older patients having been included during tool development. Given that older adults, particularly those with chronic diseases that require close monitoring, likely stand to benefit the most from improvements in quality of care that may be realized through such mHealth questionnaires, our findings suggest that future research is required to specifically identify and address age-related barriers to successful uptake of such technology. The practical barriers and statistical predictors identified in our study may be relevant for electronic questionnaire usage across settings, diseases, and patient demographics. These factors, particularly if complemented by qualitative methods to identify barriers and enablers to uptake, can be used by clinics and programs employing such questionnaires, to target improvements to the intervention and its implementation strategy in order to drive usage.

## Appendix

Multimedia Appendix 1 Supplementary tables for regression analyses of tablet refusal at first patient visit only, and refusal of the tablet after having accepted it on first occasion.

## Abbreviations

eAMS: Electronic Asthma Management System
mHealth: mobile health
OR: odds ratio
