# Supplementary material for: Primary Care Pre-Visit Electronic Patient Questionnaire for Asthma: Uptake Analysis and Predictor Modeling
Source: J Med Internet Res. 2020 Sep 18;22(9):e19358. doi: 10.2196/19358 (PMC7532461; doi:10.2196/19358)
Supplement: Multimedia Appendix 1 [file jmir_v22i9e19358_app1.doc]

**Appendix**

Table A1. Predictors of tablet refusal at first patient visit only

| **Patient-Level Descriptors** | **Visit characteristics (n= 451 visits)** | **Proportion of visits in which the tablet was refused** | **Odds Ratio^†^:**  **(95% CI)** |
| --- | --- | --- | --- |
| **Age**   - <65 years old - ≥65 years old | 357 (79.2%)  94 (20.8%) | 25/357 (7.0%)  19/94 (20.2%) | -  2.79 (1.39-5.59) |
| **Sex**   - Male - Female | 133 (29.5%)  318 (70.5%) | 11/133 (%)  33/318 (%) | -  1.37 (0.66-2.87) |
| **Current asthma medication prescription**   - No asthma medications - Any asthma medication | 112 (24.8%)  339 (75.2%) | 15/112 (13.4%)  29/339 (8.6%) | -  0.51 (0.25-1.03) |
| **Physician-documented Diagnosis of COPD**   - Absent - Present | 409 (90.7%)  42 (9.3%) | 34/409 (8.3%)  10/42 (23.8%) | -  2.53 (1.05-6.05) |
| **Clinic site**   - Site 2 - Site 1 | 111 (24.6%)  340 (75.4%) | 6/111 (5.4%)  38/340 (11.2%) | -  1.63 (0.65-4.06) |

^†^: model demonstrated good predictive ability, with a c-statistic of 0.71

Table A2. Predictors of refusing the tablet a 2nd time after having accepted it

the 1st time

| **Patient-Level Descriptors** | **Visit characteristics (n= 248 visits)** | **Proportion of visits in which the tablet was refused on 2^nd^ offering** | **Odds Ratio^†^:**  **(95% CI)** |
| --- | --- | --- | --- |
| **Age**   - <65 years old - ≥65 years old | 193 (77.8%)  55 (22.2%) | 27/193 (14.0%)  13/55 (26.3%) | -  1.93 (0.86-4.31) |
| **Sex**   - Male - Female | 68 (27.4%)  180 (72.6%) | 8/68 (11.8%)  32/180 (17.8%) | -  0.63 (0.26-1.53) |
| **Finished questionnaire when first accepted it**   - Yes - No | 221 (89.1%)  27 (10.9%) | 27/221 (12.2%)  13/27 (48.1%) | -  5.73 (2.31-14.21) |
| **Days between 1^st^ accepting tablet and being offered a 2^nd^ time (median, IQR)** | 56.0 (37.5-111.5) | 41.5 (30.0-87.0) | 1.00 (0.99-1.00) |
| **Clinic site**   - Site 2 - Site 1 | 47 (19.0%)  201 (81.0%) | 7/47 (14.9%)  33/201 (16.4%) | -  0.99 (0.36-2.71) |

^†^: model demonstrated good predictive ability, with a c-statistic of 0.75
